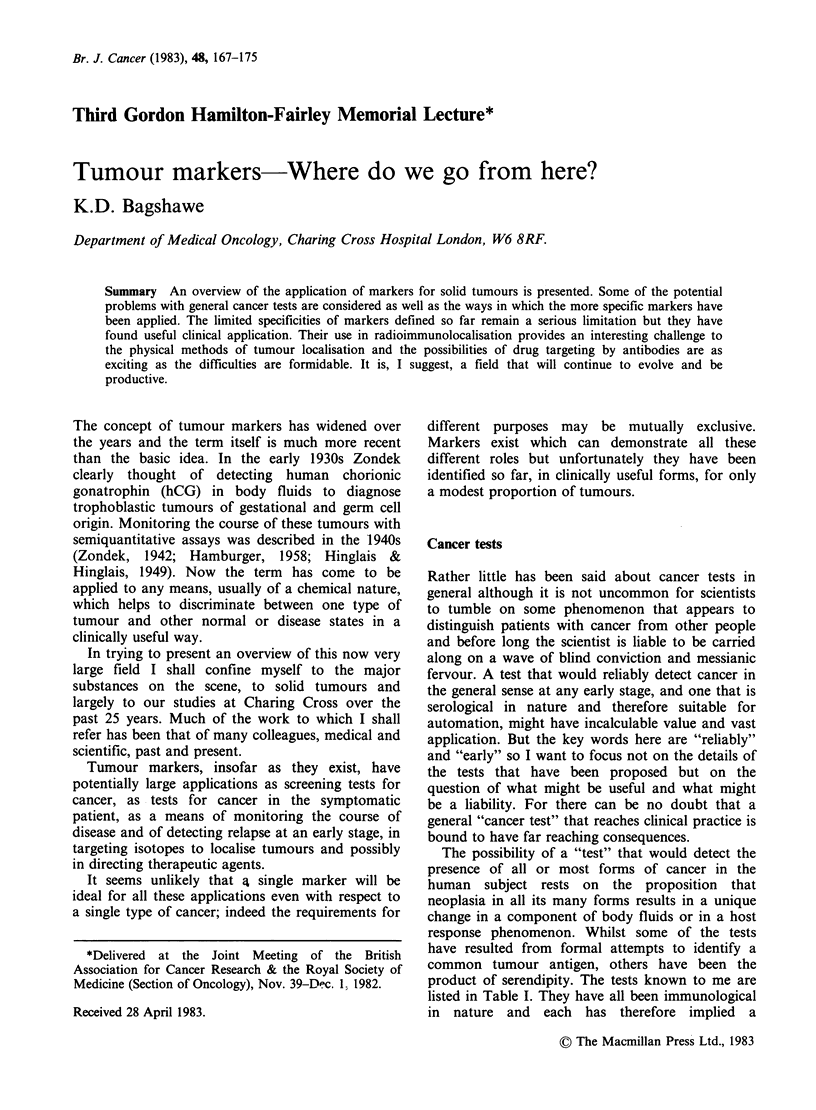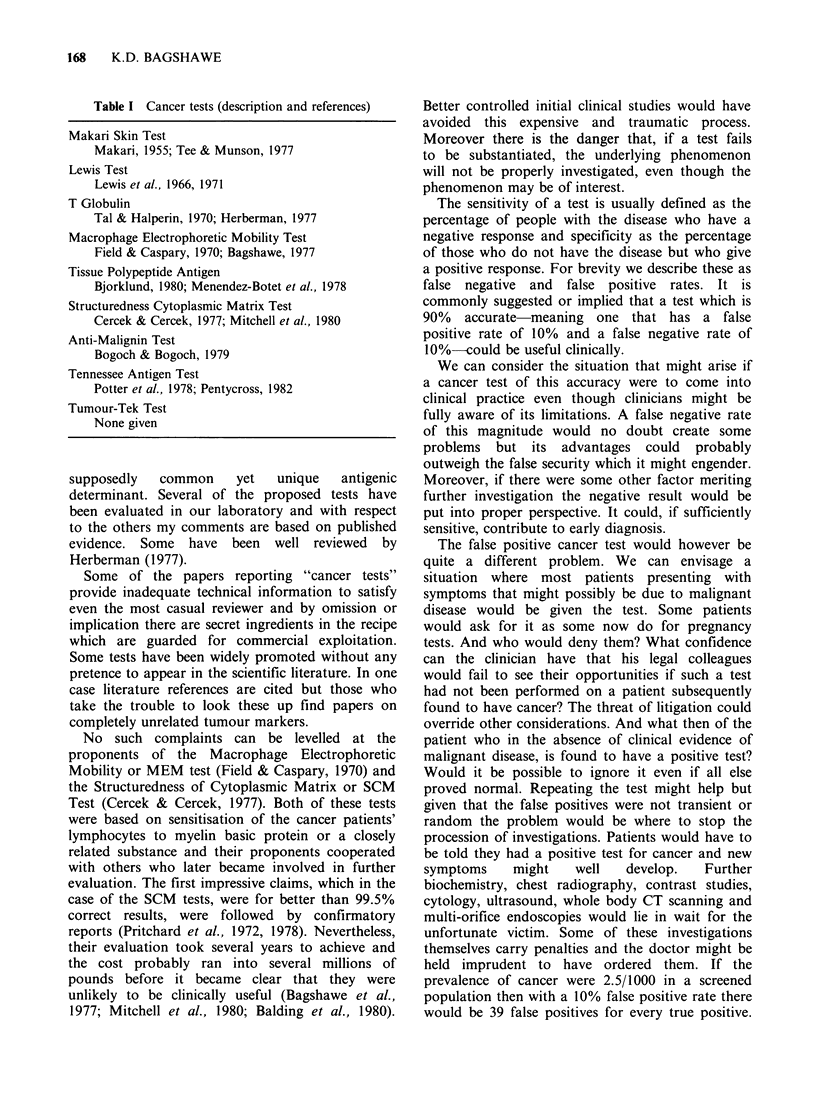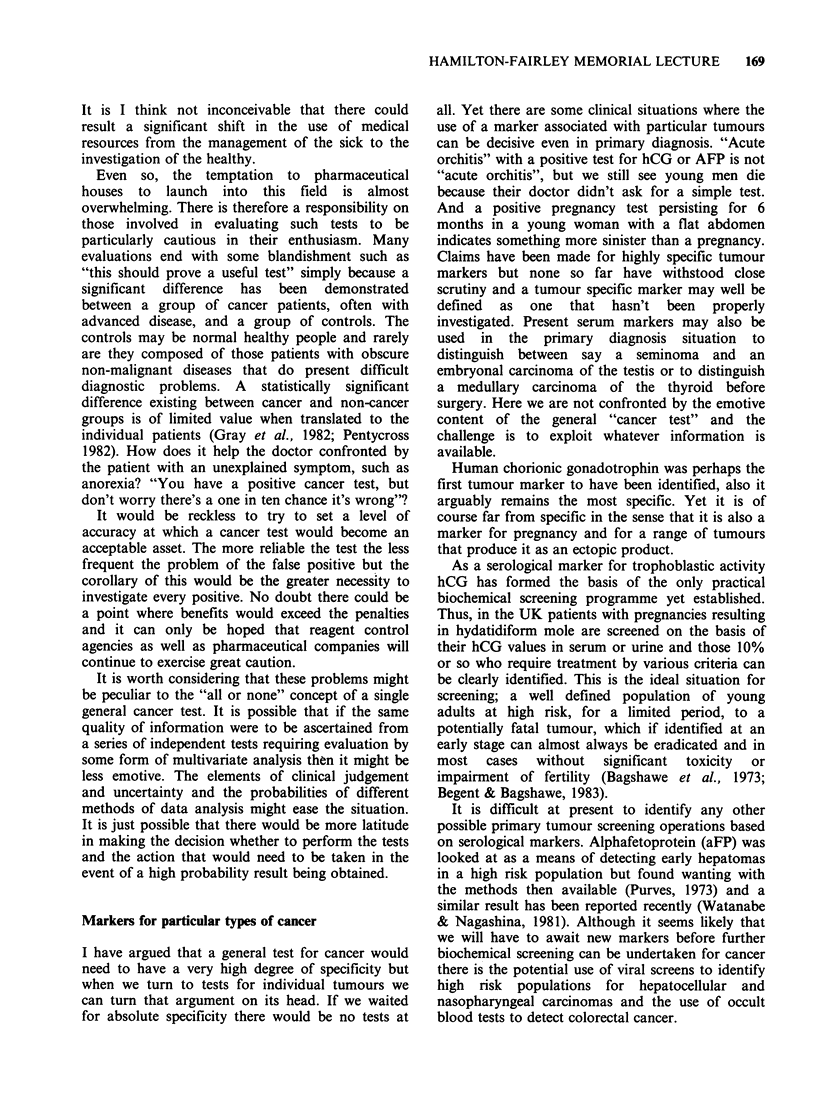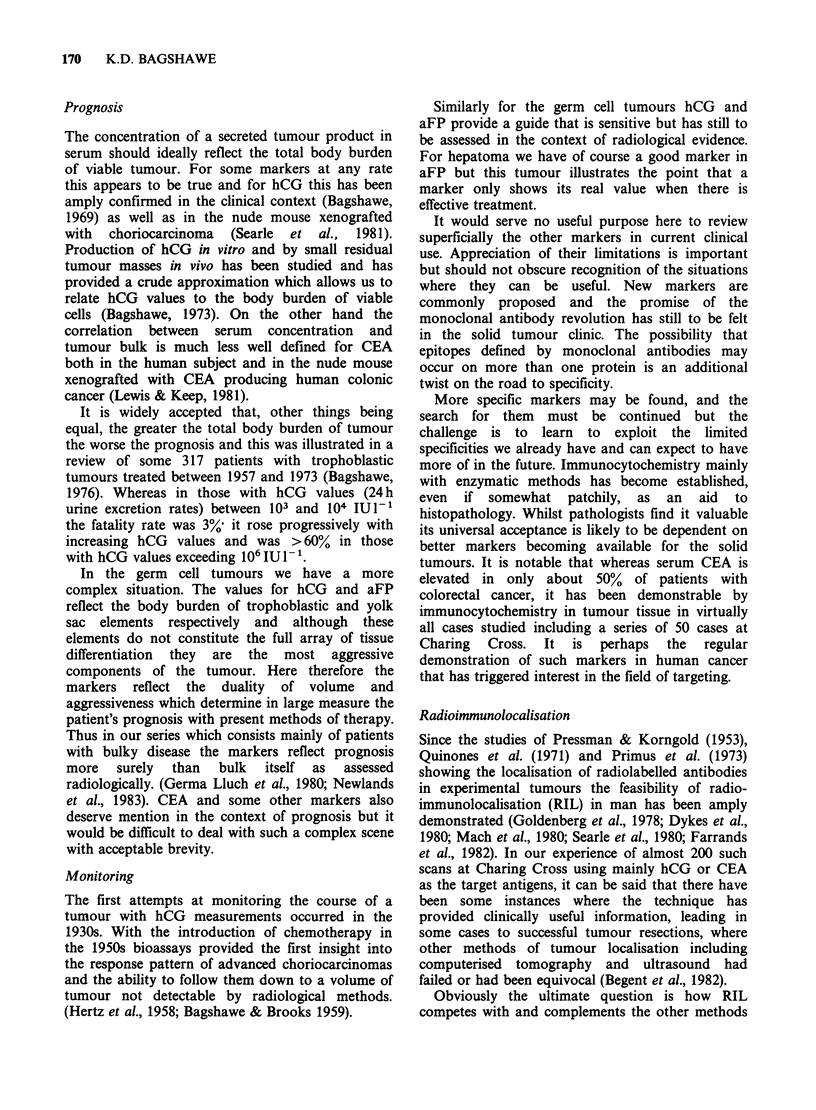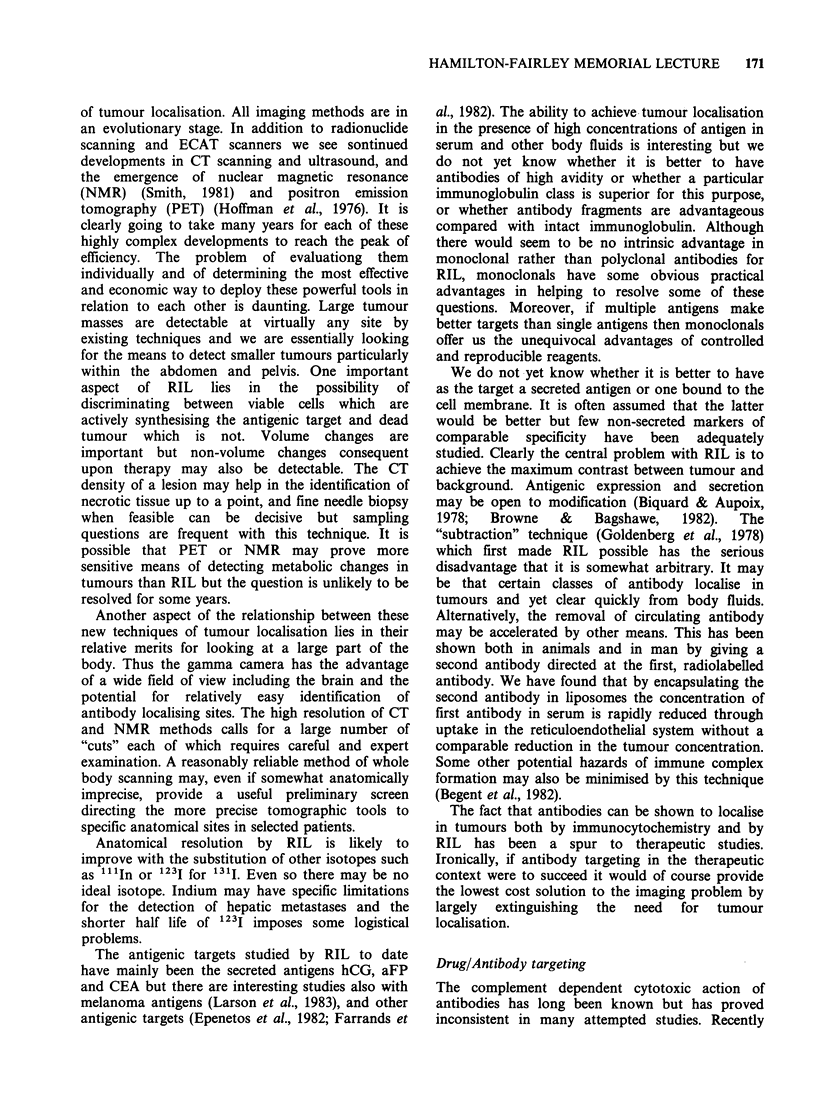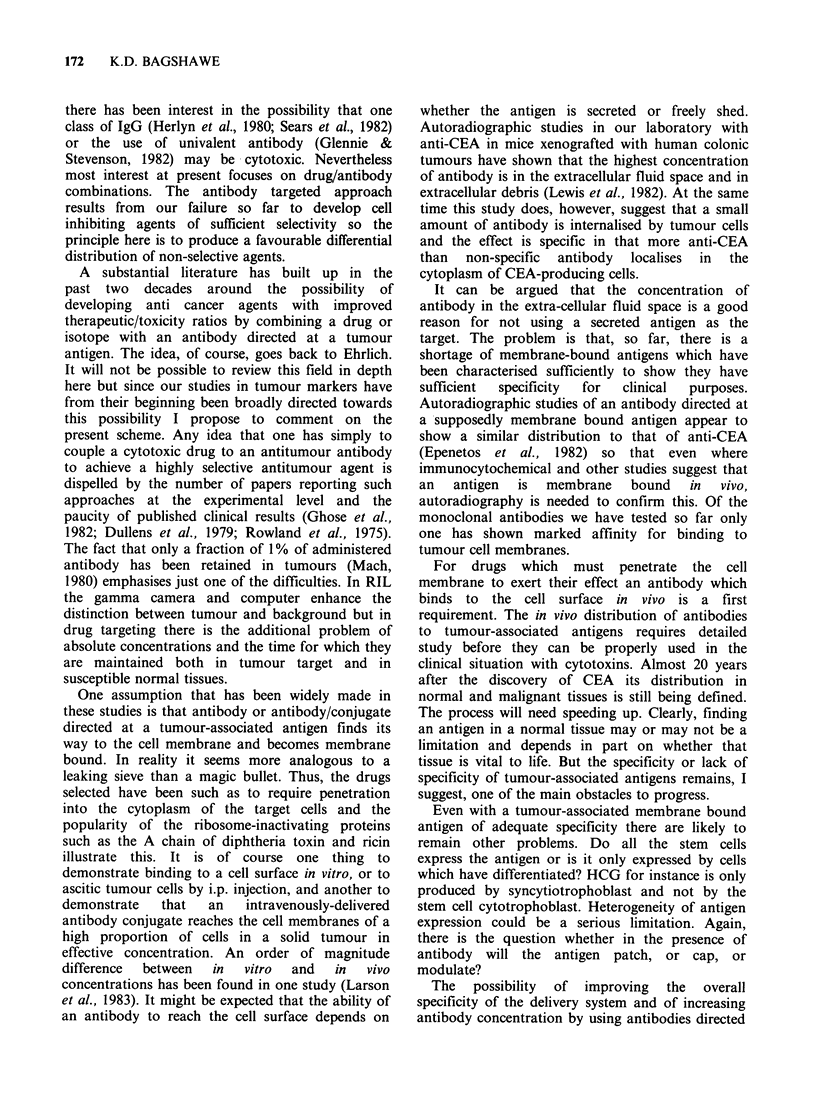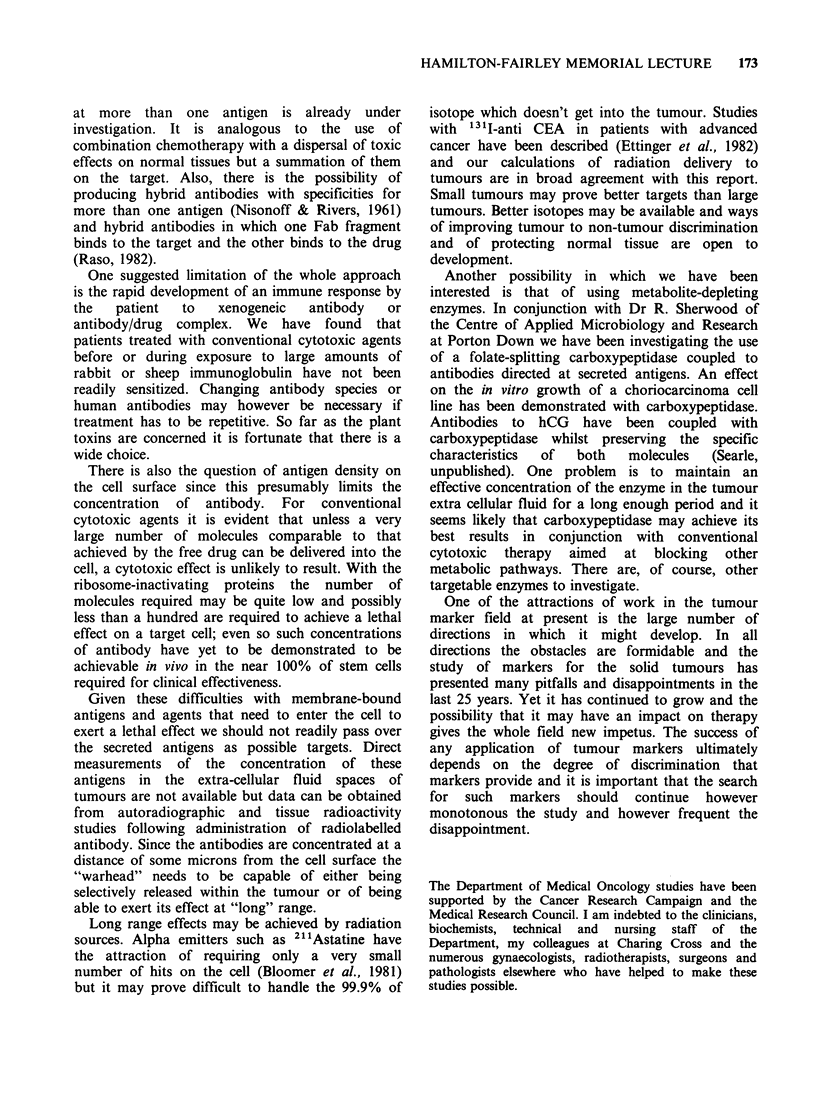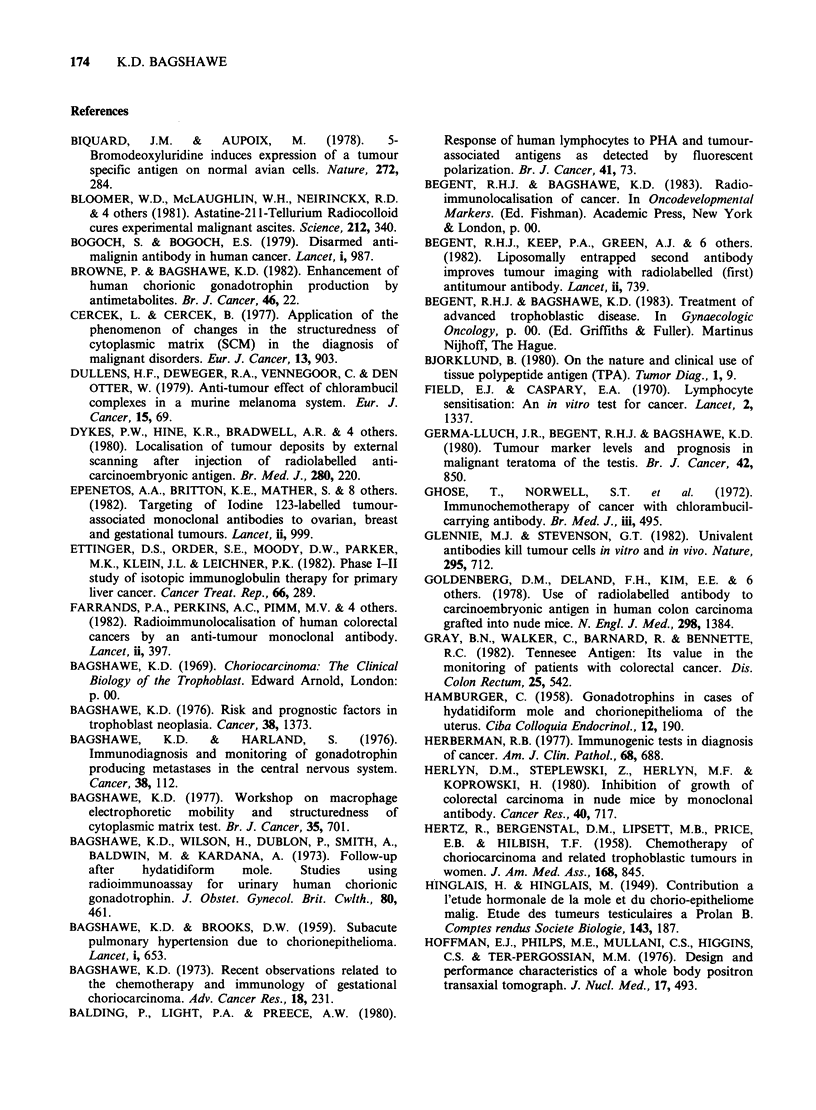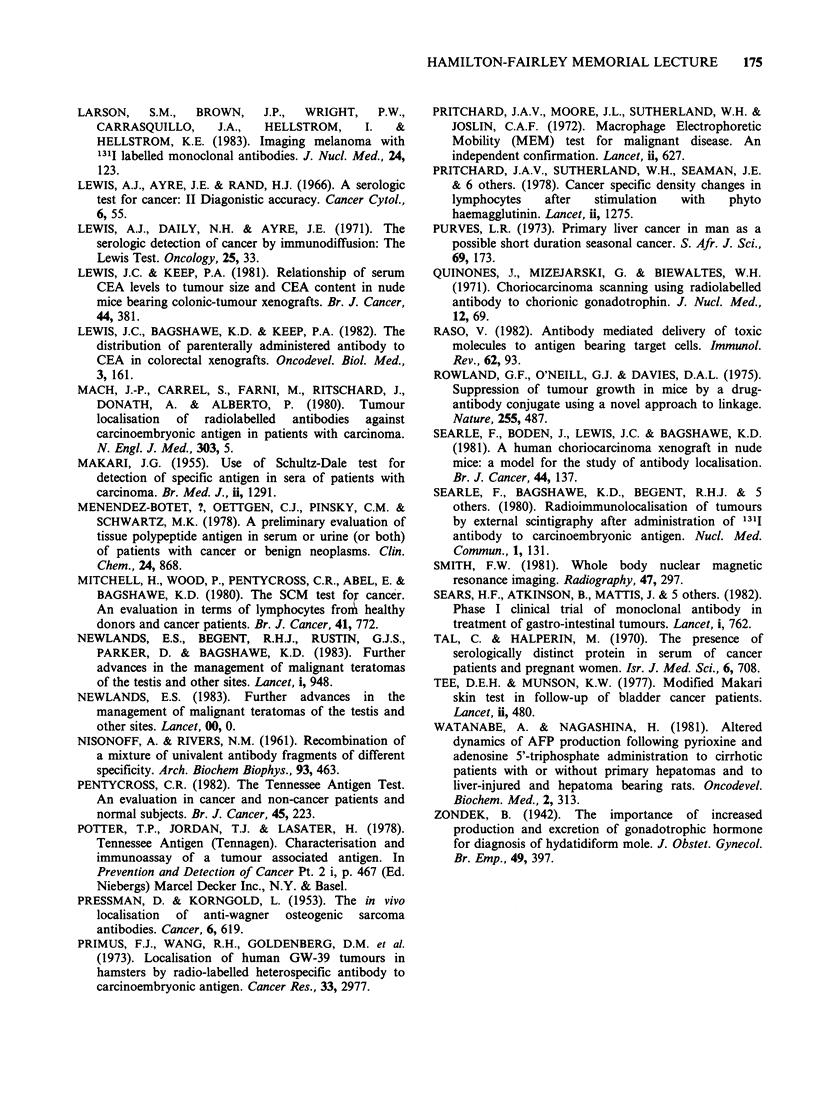# Third Gordon Hamilton-Fairley Memorial Lecture. Tumour markers--where do we go from here?

**DOI:** 10.1038/bjc.1983.172

**Published:** 1983-08

**Authors:** K. D. Bagshawe

## Abstract

An overview of the application of markers for solid tumours is presented. Some of the potential problems with general cancer tests are considered as well as the ways in which the more specific markers have been applied. The limited specificities of markers defined so far remain a serious limitation but they have found useful clinical application. Their use in radioimmunolocalisation provides an interesting challenge to the physical methods of tumour localisation and the possibilities of drug targeting by antibodies are as exciting as the difficulties are formidable. It is, I suggest, a field that will continue to evolve and be productive.


*Br. J. Cancer* (1983), **48**, 167–175

### Third Gordon Hamilton-Fairley Memorial Lecture*

### Tumour markers—Where do we go from here?

K.D. Bagshawe

*Department of Medical Oncology, Charing Cross Hospital London, W6 8RF.*

**Summary** An overview of the application of markers for solid tumours is presented. Some of the potential problems with general cancer tests are considered as well as the ways in which the more specific markers have been applied. The limited specificities of markers defined so far remain a serious limitation but they have found useful clinical application. Their use in radioimmunolocalisation provides an interesting challenge to the physical methods of tumour localisation and the possibilities of drug targeting by antibodies are as exciting as the difficulties are formidable. It is, I suggest, a field that will continue to evolve and be productive.

The concept of tumour markers has widened over the years and the term itself is much more recent than the basic idea. In the early 1930s Zondek clearly thought of detecting human chorionic gonatrophin (hCG) in body fluids to diagnose trophoblastic tumours of gestational and germ cell origin. Monitoring the course of these tumours with semiquantitative assays was described in the 1940s (Zondek, 1942; Hamburger, 1958; Hinglais & Hinglais, 1949). Now the term has come to be applied to any means, usually of a chemical nature, which helps to discriminate between one type of tumour and other normal or disease states in a clinically useful way.

In trying to present an overview of this now very large field I shall confine myself to the major substances on the scene, to solid tumours and largely to our studies at Charing Cross over the past 25 years. Much of the work to which I shall refer has been that of many colleagues, medical and scientific, past and present.

Tumour markers, insofar as they exist, have potentially large applications as screening tests for cancer, as tests for cancer in the symptomatic patient, as a means of monitoring the course of disease and of detecting relapse at an early stage, in targeting isotopes to localise tumours and possibly in directing therapeutic agents.

It seems unlikely that a single marker will be ideal for all these applications even with respect to a single type of cancer; indeed the requirements for different purposes may be mutually exclusive. Markers exist which can demonstrate all these different roles but unfortunately they have been identified so far, in clinically useful forms, for only a modest proportion of tumours.

### Cancer tests

Rather little has been said about cancer tests in general although it is not uncommon for scientists to tumble on some phenomenon that appears to distinguish patients with cancer from other people and before long the scientist is liable to be carried along on a wave of blind conviction and messianic fervour. A test that would reliably detect cancer in the general sense at any early stage, and one that is serological in nature and therefore suitable for automation, might have incalculable value and vast application. But the key words here are "reliably" and "early" so I want to focus not on the details of the tests that have been proposed but on the question of what might be useful and what might be a liability. For there can be no doubt that a general "cancer test" that reaches clinical practice is bound to have far reaching consequences.

The possibility of a "test" that would detect the presence of all or most forms of cancer in the human subject rests on the proposition that neoplasia in all its many forms results in a unique change in a component of body fluids or in a host response phenomenon. Whilst some of the tests have resulted from formal attempts to identify a common tumour antigen, others have been the product of serendipity. The tests known to me are listed in Table I. They have all been immunological in nature and each has therefore implied a

*Delivered at the Joint Meeting of the British Association for Cancer Research & the Royal Society of Medicine (Section of Oncology), Nov. 39–Dec. 1, 1982.

Received 28 April 1983.

© The Macmillan Press Ltd., 1983

**Table I** Cancer tests (description and references)

| Test | References |
|---|---|
| Makari Skin Test | Makari, 1955; Tee & Munson, 1977 |
| Lewis Test | Lewis *et al.*, 1966, 1971 |
| T Globulin | Tal & Halperin, 1970; Herberman, 1977 |
| Macrophage Electrophoretic Mobility Test | Field & Caspary, 1970; Bagshawe, 1977 |
| Tissue Polypeptide Antigen | Bjorklund, 1980; Menendez-Botet *et al.*, 1978 |
| Structuredness Cytoplasmic Matrix Test | Cercek & Cercek, 1977; Mitchell *et al.*, 1980 |
| Anti-Malignin Test | Bogoch & Bogoch, 1979 |
| Tennessee Antigen Test | Potter *et al.*, 1978; Pentycross, 1982 |
| Tumour-Tek Test | None given |

supposedly common yet unique antigenic determinant. Several of the proposed tests have been evaluated in our laboratory and with respect to the others my comments are based on published evidence. Some have been well reviewed by Herberman (1977).

Some of the papers reporting "cancer tests" provide inadequate technical information to satisfy even the most casual reviewer and by omission or implication there are secret ingredients in the recipe which are guarded for commercial exploitation. Some tests have been widely promoted without any pretence to appear in the scientific literature. In one case literature references are cited but those who take the trouble to look these up find papers on completely unrelated tumour markers.

No such complaints can be levelled at the proponents of the Macrophage Electrophoretic Mobility or MEM test (Field & Caspary, 1970) and the Structuredness of Cytoplasmic Matrix or SCM Test (Cercek & Cercek, 1977). Both of these tests were based on sensitisation of the cancer patients' lymphocytes to myelin basic protein or a closely related substance and their proponents cooperated with others who later became involved in further evaluation. The first impressive claims, which in the case of the SCM tests, were for better than 99.5% correct results, were followed by confirmatory reports (Pritchard *et al.*, 1972, 1978). Nevertheless, their evaluation took several years to achieve and the cost probably ran into several millions of pounds before it became clear that they were unlikely to be clinically useful (Bagshawe *et al.*, 1977; Mitchell *et al.*, 1980; Balding *et al.*, 1980). Better controlled initial clinical studies would have avoided this expensive and traumatic process. Moreover there is the danger that, if a test fails to be substantiated, the underlying phenomenon will not be properly investigated, even though the phenomenon may be of interest.

The sensitivity of a test is usually defined as the percentage of people with the disease who have a negative response and specificity as the percentage of those who do not have the disease but who give a positive response. For brevity we describe these as false negative and false positive rates. It is commonly suggested or implied that a test which is 90% accurate—meaning one that has a false positive rate of 10% and a false negative rate of 10%—could be useful clinically.

We can consider the situation that might arise if a cancer test of this accuracy were to come into clinical practice even though clinicians might be fully aware of its limitations. A false negative rate of this magnitude would no doubt create some problems but its advantages could probably outweigh the false security which it might engender. Moreover, if there were some other factor meriting further investigation the negative result would be put into proper perspective. It could, if sufficiently sensitive, contribute to early diagnosis.

The false positive cancer test would however be quite a different problem. We can envisage a situation where most patients presenting with symptoms that might possibly be due to malignant disease would be given the test. Some patients would ask for it as some now do for pregnancy tests. And who would deny them? What confidence can the clinician have that his legal colleagues would fail to see their opportunities if such a test had not been performed on a patient subsequently found to have cancer? The threat of litigation could override other considerations. And what then of the patient who in the absence of clinical evidence of malignant disease, is found to have a positive test? Would it be possible to ignore it even if all else proved normal. Repeating the test might help but given that the false positives were not transient or random the problem would be where to stop the procession of investigations. Patients would have to be told they had a positive test for cancer and new symptoms might well develop. Further biochemistry, chest radiography, contrast studies, cytology, ultrasound, whole body CT scanning and multi-orifice endoscopies would lie in wait for the unfortunate victim. Some of these investigations themselves carry penalties and the doctor might be held imprudent to have ordered them. If the prevalence of cancer were 2.5/1000 in a screened population then with a 10% false positive rate there would be 39 false positives for every true positive.

It is I think not inconceivable that there could result a significant shift in the use of medical resources from the management of the sick to the investigation of the healthy.

Even so, the temptation to pharmaceutical houses to launch into this field is almost overwhelming. There is therefore a responsibility on those involved in evaluating such tests to be particularly cautious in their enthusiasm. Many evaluations end with some blandishment such as "this should prove a useful test" simply because a significant difference has been demonstrated between a group of cancer patients, often with advanced disease, and a group of controls. The controls may be normal healthy people and rarely are they composed of those patients with obscure non-malignant diseases that do present difficult diagnostic problems. A statistically significant difference existing between cancer and non-cancer groups is of limited value when translated to the individual patients (Gray *et al.*, 1982; Pentycross 1982). How does it help the doctor confronted by the patient with an unexplained symptom, such as anorexia? "You have a positive cancer test, but don't worry there's a one in ten chance it's wrong"?

It would be reckless to try to set a level of accuracy at which a cancer test would become an acceptable asset. The more reliable the test the less frequent the problem of the false positive but the corollary of this would be the greater necessity to investigate every positive. No doubt there could be a point where benefits would exceed the penalties and it can only be hoped that reagent control agencies as well as pharmaceutical companies will continue to exercise great caution.

It is worth considering that these problems might be peculiar to the "all or none" concept of a single general cancer test. It is possible that if the same quality of information were to be ascertained from a series of independent tests requiring evaluation by some form of multivariate analysis then it might be less emotive. The elements of clinical judgement and uncertainty and the probabilities of different methods of data analysis might ease the situation. It is just possible that there would be more latitude in making the decision whether to perform the tests and the action that would need to be taken in the event of a high probability result being obtained.

### Markers for particular types of cancer

I have argued that a general test for cancer would need to have a very high degree of specificity but when we turn to tests for individual tumours we can turn that argument on its head. If we waited for absolute specificity there would be no tests at all. Yet there are some clinical situations where the use of a marker associated with particular tumours can be decisive even in primary diagnosis. "Acute orchitis" with a positive test for hCG or AFP is not "acute orchitis", but we still see young men die because their doctor didn't ask for a simple test. And a positive pregnancy test persisting for 6 months in a young woman with a flat abdomen indicates something more sinister than a pregnancy. Claims have been made for highly specific tumour markers but none so far have withstood close scrutiny and a tumour specific marker may well be defined as one that hasn't been properly investigated. Present serum markers may also be used in the primary diagnosis situation to distinguish between say a seminoma and an embryonal carcinoma of the testis or to distinguish a medullary carcinoma of the thyroid before surgery. Here we are not confronted by the emotive content of the general "cancer test" and the challenge is to exploit whatever information is available.

Human chorionic gonadotrophin was perhaps the first tumour marker to have been identified, also it arguably remains the most specific. Yet it is of course far from specific in the sense that it is also a marker for pregnancy and for a range of tumours that produce it as an ectopic product.

As a serological marker for trophoblastic activity hCG has formed the basis of the only practical biochemical screening programme yet established. Thus, in the UK patients with pregnancies resulting in hydatidiform mole are screened on the basis of their hCG values in serum or urine and those 10% or so who require treatment by various criteria can be clearly identified. This is the ideal situation for screening; a well defined population of young adults at high risk, for a limited period, to a potentially fatal tumour, which if identified at an early stage can almost always be eradicated and in most cases without significant toxicity or impairment of fertility (Bagshawe *et al.*, 1973; Begent & Bagshawe, 1983).

It is difficult at present to identify any other possible primary tumour screening operations based on serological markers. Alphafetoprotein (aFP) was looked at as a means of detecting early hepatomas in a high risk population but found wanting with the methods then available (Purves, 1973) and a similar result has been reported recently (Watanabe & Nagashina, 1981). Although it seems likely that we will have to await new markers before further biochemical screening can be undertaken for cancer there is the potential use of viral screens to identify high risk populations for hepatocellular and nasopharyngeal carcinomas and the use of occult blood tests to detect colorectal cancer.

*Prognosis*

The concentration of a secreted tumour product in serum should ideally reflect the total body burden of viable tumour. For some markers at any rate this appears to be true and for hCG this has been amply confirmed in the clinical context (Bagshawe, 1969) as well as in the nude mouse xenografted with choriocarcinoma (Searle *et al.*, 1981). Production of hCG *in vitro* and by small residual tumour masses *in vivo* has been studied and has provided a crude approximation which allows us to relate hCG values to the body burden of viable cells (Bagshawe, 1973). On the other hand the correlation between serum concentration and tumour bulk is much less well defined for CEA both in the human subject and in the nude mouse xenografted with CEA producing human colonic cancer (Lewis & Keep, 1981).

It is widely accepted that, other things being equal, the greater the total body burden of tumour the worse the prognosis and this was illustrated in a review of some 317 patients with trophoblastic tumours treated between 1957 and 1973 (Bagshawe, 1976). Whereas in those with hCG values (24 h urine excretion rates) between $10^3$ and $10^4$ $IU\,l^{-1}$ the fatality rate was 3%· it rose progressively with increasing hCG values and was >60% in those with hCG values exceeding $10^6\,IU\,l^{-1}$.

In the germ cell tumours we have a more complex situation. The values for hCG and aFP reflect the body burden of trophoblastic and yolk sac elements respectively and although these elements do not constitute the full array of tissue differentiation they are the most aggressive components of the tumour. Here therefore the markers reflect the duality of volume and aggressiveness which determine in large measure the patient's prognosis with present methods of therapy. Thus in our series which consists mainly of patients with bulky disease the markers reflect prognosis more surely than bulk itself as assessed radiologically. (Germa Lluch *et al.*, 1980; Newlands *et al.*, 1983). CEA and some other markers also deserve mention in the context of prognosis but it would be difficult to deal with such a complex scene with acceptable brevity.

*Monitoring*

The first attempts at monitoring the course of a tumour with hCG measurements occurred in the 1930s. With the introduction of chemotherapy in the 1950s bioassays provided the first insight into the response pattern of advanced choriocarcinomas and the ability to follow them down to a volume of tumour not detectable by radiological methods. (Hertz *et al.*, 1958; Bagshawe & Brooks 1959).

Similarly for the germ cell tumours hCG and aFP provide a guide that is sensitive but has still to be assessed in the context of radiological evidence. For hepatoma we have of course a good marker in aFP but this tumour illustrates the point that a marker only shows its real value when there is effective treatment.

It would serve no useful purpose here to review superficially the other markers in current clinical use. Appreciation of their limitations is important but should not obscure recognition of the situations where they can be useful. New markers are commonly proposed and the promise of the monoclonal antibody revolution has still to be felt in the solid tumour clinic. The possibility that epitopes defined by monoclonal antibodies may occur on more than one protein is an additional twist on the road to specificity.

More specific markers may be found, and the search for them must be continued but the challenge is to learn to exploit the limited specificities we already have and can expect to have more of in the future. Immunocytochemistry mainly with enzymatic methods has become established, even if somewhat patchily, as an aid to histopathology. Whilst pathologists find it valuable its universal acceptance is likely to be dependent on better markers becoming available for the solid tumours. It is notable that whereas serum CEA is elevated in only about 50% of patients with colorectal cancer, it has been demonstrable by immunocytochemistry in tumour tissue in virtually all cases studied including a series of 50 cases at Charing Cross. It is perhaps the regular demonstration of such markers in human cancer that has triggered interest in the field of targeting.

*Radioimmunolocalisation*

Since the studies of Pressman & Korngold (1953), Quinones *et al.* (1971) and Primus *et al.* (1973) showing the localisation of radiolabelled antibodies in experimental tumours the feasibility of radioimmunolocalisation (RIL) in man has been amply demonstrated (Goldenberg *et al.*, 1978; Dykes *et al.*, 1980; Mach *et al.*, 1980; Searle *et al.*, 1980; Farrands *et al.*, 1982). In our experience of almost 200 such scans at Charing Cross using mainly hCG or CEA as the target antigens, it can be said that there have been some instances where the technique has provided clinically useful information, leading in some cases to successful tumour resections, where other methods of tumour localisation including computerised tomography and ultrasound had failed or had been equivocal (Begent *et al.*, 1982).

Obviously the ultimate question is how RIL competes with and complements the other methods of tumour localisation. All imaging methods are in an evolutionary stage. In addition to radionuclide scanning and ECAT scanners we see sontinued developments in CT scanning and ultrasound, and the emergence of nuclear magnetic resonance (NMR) (Smith, 1981) and positron emission tomography (PET) (Hoffman *et al.*, 1976). It is clearly going to take many years for each of these highly complex developments to reach the peak of efficiency. The problem of evaluationg them individually and of determining the most effective and economic way to deploy these powerful tools in relation to each other is daunting. Large tumour masses are detectable at virtually any site by existing techniques and we are essentially looking for the means to detect smaller tumours particularly within the abdomen and pelvis. One important aspect of RIL lies in the possibility of discriminating between viable cells which are actively synthesising the antigenic target and dead tumour which is not. Volume changes are important but non-volume changes consequent upon therapy may also be detectable. The CT density of a lesion may help in the identification of necrotic tissue up to a point, and fine needle biopsy when feasible can be decisive but sampling questions are frequent with this technique. It is possible that PET or NMR may prove more sensitive means of detecting metabolic changes in tumours than RIL but the question is unlikely to be resolved for some years.

Another aspect of the relationship between these new techniques of tumour localisation lies in their relative merits for looking at a large part of the body. Thus the gamma camera has the advantage of a wide field of view including the brain and the potential for relatively easy identification of antibody localising sites. The high resolution of CT and NMR methods calls for a large number of "cuts" each of which requires careful and expert examination. A reasonably reliable method of whole body scanning may, even if somewhat anatomically imprecise, provide a useful preliminary screen directing the more precise tomographic tools to specific anatomical sites in selected patients.

Anatomical resolution by RIL is likely to improve with the substitution of other isotopes such as $^{111}$In or $^{123}$I for $^{131}$I. Even so there may be no ideal isotope. Indium may have specific limitations for the detection of hepatic metastases and the shorter half life of $^{123}$I imposes some logistical problems.

The antigenic targets studied by RIL to date have mainly been the secreted antigens hCG, aFP and CEA but there are interesting studies also with melanoma antigens (Larson *et al.*, 1983), and other antigenic targets (Epenetos *et al.*, 1982; Farrands *et al.*, 1982). The ability to achieve tumour localisation in the presence of high concentrations of antigen in serum and other body fluids is interesting but we do not yet know whether it is better to have antibodies of high avidity or whether a particular immunoglobulin class is superior for this purpose, or whether antibody fragments are advantageous compared with intact immunoglobulin. Although there would seem to be no intrinsic advantage in monoclonal rather than polyclonal antibodies for RIL, monoclonals have some obvious practical advantages in helping to resolve some of these questions. Moreover, if multiple antigens make better targets than single antigens then monoclonals offer us the unequivocal advantages of controlled and reproducible reagents.

We do not yet know whether it is better to have as the target a secreted antigen or one bound to the cell membrane. It is often assumed that the latter would be better but few non-secreted markers of comparable specificity have been adequately studied. Clearly the central problem with RIL is to achieve the maximum contrast between tumour and background. Antigenic expression and secretion may be open to modification (Biquard & Aupoix, 1978; Browne & Bagshawe, 1982). The "subtraction" technique (Goldenberg *et al.*, 1978) which first made RIL possible has the serious disadvantage that it is somewhat arbitrary. It may be that certain classes of antibody localise in tumours and yet clear quickly from body fluids. Alternatively, the removal of circulating antibody may be accelerated by other means. This has been shown both in animals and in man by giving a second antibody directed at the first, radiolabelled antibody. We have found that by encapsulating the second antibody in liposomes the concentration of first antibody in serum is rapidly reduced through uptake in the reticuloendothelial system without a comparable reduction in the tumour concentration. Some other potential hazards of immune complex formation may also be minimised by this technique (Begent *et al.*, 1982).

The fact that antibodies can be shown to localise in tumours both by immunocytochemistry and by RIL has been a spur to therapeutic studies. Ironically, if antibody targeting in the therapeutic context were to succeed it would of course provide the lowest cost solution to the imaging problem by largely extinguishing the need for tumour localisation.

### *Drug/Antibody targeting*

The complement dependent cytotoxic action of antibodies has long been known but has proved inconsistent in many attempted studies. Recently there has been interest in the possibility that one class of IgG (Herlyn *et al.*, 1980; Sears *et al.*, 1982) or the use of univalent antibody (Glennie & Stevenson, 1982) may be cytotoxic. Nevertheless most interest at present focuses on drug/antibody combinations. The antibody targeted approach results from our failure so far to develop cell inhibiting agents of sufficient selectivity so the principle here is to produce a favourable differential distribution of non-selective agents.

A substantial literature has built up in the past two decades around the possibility of developing anti cancer agents with improved therapeutic/toxicity ratios by combining a drug or isotope with an antibody directed at a tumour antigen. The idea, of course, goes back to Ehrlich. It will not be possible to review this field in depth here but since our studies in tumour markers have from their beginning been broadly directed towards this possibility I propose to comment on the present scheme. Any idea that one has simply to couple a cytotoxic drug to an antitumour antibody to achieve a highly selective antitumour agent is dispelled by the number of papers reporting such approaches at the experimental level and the paucity of published clinical results (Ghose *et al.*, 1982; Dullens *et al.*, 1979; Rowland *et al.*, 1975). The fact that only a fraction of 1% of administered antibody has been retained in tumours (Mach, 1980) emphasises just one of the difficulties. In RIL the gamma camera and computer enhance the distinction between tumour and background but in drug targeting there is the additional problem of absolute concentrations and the time for which they are maintained both in tumour target and in susceptible normal tissues.

One assumption that has been widely made in these studies is that antibody or antibody/conjugate directed at a tumour-associated antigen finds its way to the cell membrane and becomes membrane bound. In reality it seems more analogous to a leaking sieve than a magic bullet. Thus, the drugs selected have been such as to require penetration into the cytoplasm of the target cells and the popularity of the ribosome-inactivating proteins such as the A chain of diphtheria toxin and ricin illustrate this. It is of course one thing to demonstrate binding to a cell surface *in vitro*, or to ascitic tumour cells by i.p. injection, and another to demonstrate that an intravenously-delivered antibody conjugate reaches the cell membranes of a high proportion of cells in a solid tumour in effective concentration. An order of magnitude difference between *in vitro* and *in vivo* concentrations has been found in one study (Larson *et al.*, 1983). It might be expected that the ability of an antibody to reach the cell surface depends on whether the antigen is secreted or freely shed. Autoradiographic studies in our laboratory with anti-CEA in mice xenografted with human colonic tumours have shown that the highest concentration of antibody is in the extracellular fluid space and in extracellular debris (Lewis *et al.*, 1982). At the same time this study does, however, suggest that a small amount of antibody is internalised by tumour cells and the effect is specific in that more anti-CEA than non-specific antibody localises in the cytoplasm of CEA-producing cells.

It can be argued that the concentration of antibody in the extra-cellular fluid space is a good reason for not using a secreted antigen as the target. The problem is that, so far, there is a shortage of membrane-bound antigens which have been characterised sufficiently to show they have sufficient specificity for clinical purposes. Autoradiographic studies of an antibody directed at a supposedly membrane bound antigen appear to show a similar distribution to that of anti-CEA (Epenetos *et al.*, 1982) so that even where immunocytochemical and other studies suggest that an antigen is membrane bound *in vivo*, autoradiography is needed to confirm this. Of the monoclonal antibodies we have tested so far only one has shown marked affinity for binding to tumour cell membranes.

For drugs which must penetrate the cell membrane to exert their effect an antibody which binds to the cell surface *in vivo* is a first requirement. The *in vivo* distribution of antibodies to tumour-associated antigens requires detailed study before they can be properly used in the clinical situation with cytotoxins. Almost 20 years after the discovery of CEA its distribution in normal and malignant tissues is still being defined. The process will need speeding up. Clearly, finding an antigen in a normal tissue may or may not be a limitation and depends in part on whether that tissue is vital to life. But the specificity or lack of specificity of tumour-associated antigens remains, I suggest, one of the main obstacles to progress.

Even with a tumour-associated membrane bound antigen of adequate specificity there are likely to remain other problems. Do all the stem cells express the antigen or is it only expressed by cells which have differentiated? HCG for instance is only produced by syncytiotrophoblast and not by the stem cell cytotrophoblast. Heterogeneity of antigen expression could be a serious limitation. Again, there is the question whether in the presence of antibody will the antigen patch, or cap, or modulate?

The possibility of improving the overall specificity of the delivery system and of increasing antibody concentration by using antibodies directed at more than one antigen is already under investigation. It is analogous to the use of combination chemotherapy with a dispersal of toxic effects on normal tissues but a summation of them on the target. Also, there is the possibility of producing hybrid antibodies with specificities for more than one antigen (Nisonoff & Rivers, 1961) and hybrid antibodies in which one Fab fragment binds to the target and the other binds to the drug (Raso, 1982).

One suggested limitation of the whole approach is the rapid development of an immune response by the patient to xenogeneic antibody or antibody/drug complex. We have found that patients treated with conventional cytotoxic agents before or during exposure to large amounts of rabbit or sheep immunoglobulin have not been readily sensitized. Changing antibody species or human antibodies may however be necessary if treatment has to be repetitive. So far as the plant toxins are concerned it is fortunate that there is a wide choice.

There is also the question of antigen density on the cell surface since this presumably limits the concentration of antibody. For conventional cytotoxic agents it is evident that unless a very large number of molecules comparable to that achieved by the free drug can be delivered into the cell, a cytotoxic effect is unlikely to result. With the ribosome-inactivating proteins the number of molecules required may be quite low and possibly less than a hundred are required to achieve a lethal effect on a target cell; even so such concentrations of antibody have yet to be demonstrated to be achievable *in vivo* in the near 100% of stem cells required for clinical effectiveness.

Given these difficulties with membrane-bound antigens and agents that need to enter the cell to exert a lethal effect we should not readily pass over the secreted antigens as possible targets. Direct measurements of the concentration of these antigens in the extra-cellular fluid spaces of tumours are not available but data can be obtained from autoradiographic and tissue radioactivity studies following administration of radiolabelled antibody. Since the antibodies are concentrated at a distance of some microns from the cell surface the "warhead" needs to be capable of either being selectively released within the tumour or of being able to exert its effect at "long" range.

Long range effects may be achieved by radiation sources. Alpha emitters such as $^{211}$Astatine have the attraction of requiring only a very small number of hits on the cell (Bloomer *et al.*, 1981) but it may prove difficult to handle the 99.9% of isotope which doesn't get into the tumour. Studies with $^{131}$I-anti CEA in patients with advanced cancer have been described (Ettinger *et al.*, 1982) and our calculations of radiation delivery to tumours are in broad agreement with this report. Small tumours may prove better targets than large tumours. Better isotopes may be available and ways of improving tumour to non-tumour discrimination and of protecting normal tissue are open to development.

Another possibility in which we have been interested is that of using metabolite-depleting enzymes. In conjunction with Dr R. Sherwood of the Centre of Applied Microbiology and Research at Porton Down we have been investigating the use of a folate-splitting carboxypeptidase coupled to antibodies directed at secreted antigens. An effect on the *in vitro* growth of a choriocarcinoma cell line has been demonstrated with carboxypeptidase. Antibodies to hCG have been coupled with carboxypeptidase whilst preserving the specific characteristics of both molecules (Searle, unpublished). One problem is to maintain an effective concentration of the enzyme in the tumour extra cellular fluid for a long enough period and it seems likely that carboxypeptidase may achieve its best results in conjunction with conventional cytotoxic therapy aimed at blocking other metabolic pathways. There are, of course, other targetable enzymes to investigate.

One of the attractions of work in the tumour marker field at present is the large number of directions in which it might develop. In all directions the obstacles are formidable and the study of markers for the solid tumours has presented many pitfalls and disappointments in the last 25 years. Yet it has continued to grow and the possibility that it may have an impact on therapy gives the whole field new impetus. The success of any application of tumour markers ultimately depends on the degree of discrimination that markers provide and it is important that the search for such markers should continue however monotonous the study and however frequent the disappointment.

The Department of Medical Oncology studies have been supported by the Cancer Research Campaign and the Medical Research Council. I am indebted to the clinicians, biochemists, technical and nursing staff of the Department, my colleagues at Charing Cross and the numerous gynaecologists, radiotherapists, surgeons and pathologists elsewhere who have helped to make these studies possible.

### References

BIQUARD, J.M. & AUPOIX, M. (1978). 5-Bromodeoxyluridine induces expression of a tumour specific antigen on normal avian cells. *Nature*, **272**, 284.

BLOOMER, W.D., McLAUGHLIN, W.H., NEIRINCKX, R.D. & 4 others (1981). Astatine-211-Tellurium Radiocolloid cures experimental malignant ascites. *Science*, **212**, 340.

BOGOCH, S. & BOGOCH, E.S. (1979). Disarmed anti-malignin antibody in human cancer. *Lancet*, **i**, 987.

BROWNE, P. & BAGSHAWE, K.D. (1982). Enhancement of human chorionic gonadotrophin production by antimetabolites. *Br. J. Cancer*, **46**, 22.

CERCEK, L. & CERCEK, B. (1977). Application of the phenomenon of changes in the structuredness of cytoplasmic matrix (SCM) in the diagnosis of malignant disorders. *Eur. J. Cancer*, **13**, 903.

DULLENS, H.F., DEWEGER, R.A., VENNEGOOR, C. & DEN OTTER, W. (1979). Anti-tumour effect of chlorambucil complexes in a murine melanoma system. *Eur. J. Cancer*, **15**, 69.

DYKES, P.W., HINE, K.R., BRADWELL, A.R. & 4 others. (1980). Localisation of tumour deposits by external scanning after injection of radiolabelled anti-carcinoembryonic antigen. *Br. Med. J.*, **280**, 220.

EPENETOS, A.A., BRITTON, K.E., MATHER, S. & 8 others. (1982). Targeting of Iodine 123-labelled tumour-associated monoclonal antibodies to ovarian, breast and gestational tumours. *Lancet*, **ii**, 999.

ETTINGER, D.S., ORDER, S.E., MOODY, D.W., PARKER, M.K., KLEIN, J.L. & LEICHNER, P.K. (1982). Phase I–II study of isotopic immunoglobulin therapy for primary liver cancer. *Cancer Treat. Rep.*, **66**, 289.

FARRANDS, P.A., PERKINS, A.C., PIMM, M.V. & 4 others. (1982). Radioimmunolocalisation of human colorectal cancers by an anti-tumour monoclonal antibody. *Lancet*, **ii**, 397.

BAGSHAWE, K.D. (1969). *Choriocarcinoma: The Clinical Biology of the Trophoblast*. Edward Arnold, London: p. 00.

BAGSHAWE, K.D. (1976). Risk and prognostic factors in trophoblast neoplasia. *Cancer*, **38**, 1373.

BAGSHAWE, K.D. & HARLAND, S. (1976). Immunodiagnosis and monitoring of gonadotrophin producing metastases in the central nervous system. *Cancer*, **38**, 112.

BAGSHAWE, K.D. (1977). Workshop on macrophage electrophoretic mobility and structuredness of cytoplasmic matrix test. *Br. J. Cancer*, **35**, 701.

BAGSHAWE, K.D., WILSON, H., DUBLON, P., SMITH, A., BALDWIN, M. & KARDANA, A. (1973). Follow-up after hydatidiform mole. Studies using radioimmunoassay for urinary human chorionic gonadotrophin. *J. Obstet. Gynecol. Brit. Cwlth.*, **80**, 461.

BAGSHAWE, K.D. & BROOKS, D.W. (1959). Subacute pulmonary hypertension due to chorionepithelioma. *Lancet*, **i**, 653.

BAGSHAWE, K.D. (1973). Recent observations related to the chemotherapy and immunology of gestational choriocarcinoma. *Adv. Cancer Res.*, **18**, 231.

BALDING, P., LIGHT, P.A. & PREECE, A.W. (1980). Response of human lymphocytes to PHA and tumour-associated antigens as detected by fluorescent polarization. *Br. J. Cancer*, **41**, 73.

BEGENT, R.H.J. & BAGSHAWE, K.D. (1983). Radio-immunolocalisation of cancer. In *Oncodevelopmental Markers*. (Ed. Fishman). Academic Press, New York & London, p. 00.

BEGENT, R.H.J., KEEP, P.A., GREEN, A.J. & 6 others. (1982). Liposomally entrapped second antibody improves tumour imaging with radiolabelled (first) antitumour antibody. *Lancet*, **ii**, 739.

BEGENT, R.H.J. & BAGSHAWE, K.D. (1983). Treatment of advanced trophoblastic disease. In *Gynaecologic Oncology*, p. 00. (Ed. Griffiths & Fuller). Martinus Nijhoff, The Hague.

BJORKLUND, B. (1980). On the nature and clinical use of tissue polypeptide antigen (TPA). *Tumor Diag.*, **1**, 9.

FIELD, E.J. & CASPARY, E.A. (1970). Lymphocyte sensitisation: An *in vitro* test for cancer. *Lancet*, **2**, 1337.

GERMA-LLUCH, J.R., BEGENT, R.H.J. & BAGSHAWE, K.D. (1980). Tumour marker levels and prognosis in malignant teratoma of the testis. *Br. J. Cancer*, **42**, 850.

GHOSE, T., NORWELL, S.T. *et al.* (1972). Immunochemotherapy of cancer with chlorambucil-carrying antibody. *Br. Med. J.*, **iii**, 495.

GLENNIE, M.J. & STEVENSON, G.T. (1982). Univalent antibodies kill tumour cells *in vitro* and *in vivo*. *Nature*, **295**, 712.

GOLDENBERG, D.M., DELAND, F.H., KIM, E.E. & 6 others. (1978). Use of radiolabelled antibody to carcinoembryonic antigen in human colon carcinoma grafted into nude mice. *N. Engl. J. Med.*, **298**, 1384.

GRAY, B.N., WALKER, C., BARNARD, R. & BENNETTE, R.C. (1982). Tennesee Antigen: Its value in the monitoring of patients with colorectal cancer. *Dis. Colon Rectum*, **25**, 542.

HAMBURGER, C. (1958). Gonadotrophins in cases of hydatidiform mole and chorionepithelioma of the uterus. *Ciba Colloquia Endocrinol.*, **12**, 190.

HERBERMAN, R.B. (1977). Immunogenic tests in diagnosis of cancer. *Am. J. Clin. Pathol.*, **68**, 688.

HERLYN, D.M., STEPLEWSKI, Z., HERLYN, M.F. & KOPROWSKI, H. (1980). Inhibition of growth of colorectal carcinoma in nude mice by monoclonal antibody. *Cancer Res.*, **40**, 717.

HERTZ, R., BERGENSTAL, D.M., LIPSETT, M.B., PRICE, E.B. & HILBISH, T.F. (1958). Chemotherapy of choriocarcinoma and related trophoblastic tumours in women. *J. Am. Med. Ass.*, **168**, 845.

HINGLAIS, H. & HINGLAIS, M. (1949). Contribution a l'etude hormonale de la mole et du chorio-epitheliome malig. Etude des tumeurs testiculaires a Prolan B. *Comptes rendus Societe Biologie*, **143**, 187.

HOFFMAN, E.J., PHILPS, M.E., MULLANI, C.S., HIGGINS, C.S. & TER-PERGOSSIAN, M.M. (1976). Design and performance characteristics of a whole body positron transaxial tomograph. *J. Nucl. Med.*, **17**, 493.

LARSON, S.M., BROWN, J.P., WRIGHT, P.W., CARRASQUILLO, J.A., HELLSTROM, I. & HELLSTROM, K.E. (1983). Imaging melanoma with $^{131}$I labelled monoclonal antibodies. *J. Nucl. Med.*, **24**, 123.

LEWIS, A.J., AYRE, J.E. & RAND, H.J. (1966). A serologic test for cancer: II Diagonistic accuracy. *Cancer Cytol.*, **6**, 55.

LEWIS, A.J., DAILY, N.H. & AYRE, J.E. (1971). The serologic detection of cancer by immunodiffusion: The Lewis Test. *Oncology*, **25**, 33.

LEWIS, J.C. & KEEP, P.A. (1981). Relationship of serum CEA levels to tumour size and CEA content in nude mice bearing colonic-tumour xenografts. *Br. J. Cancer*, **44**, 381.

LEWIS, J.C., BAGSHAWE, K.D. & KEEP, P.A. (1982). The distribution of parenterally administered antibody to CEA in colorectal xenografts. *Oncodevel. Biol. Med.*, **3**, 161.

MACH, J.-P., CARREL, S., FARNI, M., RITSCHARD, J., DONATH, A. & ALBERTO, P. (1980). Tumour localisation of radiolabelled antibodies against carcinoembryonic antigen in patients with carcinoma. *N. Engl. J. Med.*, **303**, 5.

MAKARI, J.G. (1955). Use of Schultz-Dale test for detection of specific antigen in sera of patients with carcinoma. *Br. Med. J.*, **ii**, 1291.

MENENDEZ-BOTET, ?, OETTGEN, C.J., PINSKY, C.M. & SCHWARTZ, M.K. (1978). A preliminary evaluation of tissue polypeptide antigen in serum or urine (or both) of patients with cancer or benign neoplasms. *Clin. Chem.*, **24**, 868.

MITCHELL, H., WOOD, P., PENTYCROSS, C.R., ABEL, E. & BAGSHAWE, K.D. (1980). The SCM test for cancer. An evaluation in terms of lymphocytes from healthy donors and cancer patients. *Br. J. Cancer*, **41**, 772.

NEWLANDS, E.S., BEGENT, R.H.J., RUSTIN, G.J.S., PARKER, D. & BAGSHAWE, K.D. (1983). Further advances in the management of malignant teratomas of the testis and other sites. *Lancet*, **i**, 948.

NEWLANDS, E.S. (1983). Further advances in the management of malignant teratomas of the testis and other sites. *Lancet*, **00**, 0.

NISONOFF, A. & RIVERS, N.M. (1961). Recombination of a mixture of univalent antibody fragments of different specificity. *Arch. Biochem Biophys.*, **93**, 463.

PENTYCROSS, C.R. (1982). The Tennessee Antigen Test. An evaluation in cancer and non-cancer patients and normal subjects. *Br. J. Cancer*, **45**, 223.

POTTER, T.P., JORDAN, T.J. & LASATER, H. (1978). Tennessee Antigen (Tennagen). Characterisation and immunoassay of a tumour associated antigen. In *Prevention and Detection of Cancer* Pt. 2 i, p. 467 (Ed. Niebergs) Marcel Decker Inc., N.Y. & Basel.

PRESSMAN, D. & KORNGOLD, L. (1953). The *in vivo* localisation of anti-wagner osteogenic sarcoma antibodies. *Cancer*, **6**, 619.

PRIMUS, F.J., WANG, R.H., GOLDENBERG, D.M. *et al.* (1973). Localisation of human GW-39 tumours in hamsters by radio-labelled heterospecific antibody to carcinoembryonic antigen. *Cancer Res.*, **33**, 2977.

PRITCHARD, J.A.V., MOORE, J.L., SUTHERLAND, W.H. & JOSLIN, C.A.F. (1972). Macrophage Electrophoretic Mobility (MEM) test for malignant disease. An independent confirmation. *Lancet*, **ii**, 627.

PRITCHARD, J.A.V., SUTHERLAND, W.H., SEAMAN, J.E. & 6 others. (1978). Cancer specific density changes in lymphocytes after stimulation with phyto haemagglutinin. *Lancet*, **ii**, 1275.

PURVES, L.R. (1973). Primary liver cancer in man as a possible short duration seasonal cancer. *S. Afr. J. Sci.*, **69**, 173.

QUINONES, J., MIZEJARSKI, G. & BIEWALTES, W.H. (1971). Choriocarcinoma scanning using radiolabelled antibody to chorionic gonadotrophin. *J. Nucl. Med.*, **12**, 69.

RASO, V. (1982). Antibody mediated delivery of toxic molecules to antigen bearing target cells. *Immunol. Rev.*, **62**, 93.

ROWLAND, G.F., O'NEILL, G.J. & DAVIES, D.A.L. (1975). Suppression of tumour growth in mice by a drug-antibody conjugate using a novel approach to linkage. *Nature*, **255**, 487.

SEARLE, F., BODEN, J., LEWIS, J.C. & BAGSHAWE, K.D. (1981). A human choriocarcinoma xenograft in nude mice: a model for the study of antibody localisation. *Br. J. Cancer*, **44**, 137.

SEARLE, F., BAGSHAWE, K.D., BEGENT, R.H.J. & 5 others. (1980). Radioimmunolocalisation of tumours by external scintigraphy after administration of $^{131}$I antibody to carcinoembryonic antigen. *Nucl. Med. Commun.*, **1**, 131.

SMITH, F.W. (1981). Whole body nuclear magnetic resonance imaging. *Radiography*, **47**, 297.

SEARS, H.F., ATKINSON, B., MATTIS, J. & 5 others. (1982). Phase I clinical trial of monoclonal antibody in treatment of gastro-intestinal tumours. *Lancet*, **i**, 762.

TAL, C. & HALPERIN, M. (1970). The presence of serologically distinct protein in serum of cancer patients and pregnant women. *Isr. J. Med. Sci.*, **6**, 708.

TEE, D.E.H. & MUNSON, K.W. (1977). Modified Makari skin test in follow-up of bladder cancer patients. *Lancet*, **ii**, 480.

WATANABE, A. & NAGASHINA, H. (1981). Altered dynamics of AFP production following pyrioxine and adenosine 5'-triphosphate administration to cirrhotic patients with or without primary hepatomas and to liver-injured and hepatoma bearing rats. *Oncodevel. Biochem. Med.*, **2**, 313.

ZONDEK, B. (1942). The importance of increased production and excretion of gonadotrophic hormone for diagnosis of hydatidiform mole. *J. Obstet. Gynecol. Br. Emp.*, **49**, 397.